# Impact of Self-Efficacy and Self-Esteem on Caregiver Burden in Formal and Informal Caregivers of People with Dependency: A Systematic Review

**DOI:** 10.3390/healthcare14152267

**Published:** 2026-07-24

**Authors:** Faustino Andrés-Pérez, Lluna Maria Bru-Luna, Sergio Hidalgo-Fuentes, Manuel Martí-Vilar

**Affiliations:** 1 Department of Basic Psychology, Faculty of Psychology and Speech Therapy, Universitat de València, 46010 Valencia, Spain; fausanpe@alumni.uv.es (F.A.-P.); sergio.hidalgo@uv.es (S.H.-F.); 2 Department of Education, Faculty of Social Sciences, Universidad Europea de Valencia, 46010 Valencia, Spain; lluna.bru@universidadeuropea.es

**Keywords:** self-efficacy, self-esteem, caregivers, overload, disability, mental health, resilience, well-being

## Abstract

**Background/Objectives:** Although self-efficacy and self-esteem have been associated with caregiver well-being, the available evidence remains fragmented across different caregiving populations and outcomes. Therefore, this systematic review aimed to synthesize the available scientific evidence on the relationship between self-efficacy, self-esteem, and caregiver burden among formal and informal caregivers of dependent individuals, providing an integrative framework to clarify the protective role of these psychological resources. **Methods**: A systematic review was conducted following PRISMA guidelines, including fifty-six studies of medium and high methodological quality identified through exhaustive searches of international databases up to March 2026. **Results**: The evidence shows a consistent negative association between self-efficacy and caregiver burden, indicating that greater confidence in caregiving abilities is linked to lower perceived stress and better quality of life among caregivers of people with disabilities and dependency, while self-esteem, although less frequently studied, is associated with higher resilience, lower emotional vulnerability, and more adaptive responses to caregiving demands. **Conclusions**: From a public health and disability perspective, these findings underscore the central role of psychological resources in caregiver outcomes and suggest that interventions designed to strengthen self-efficacy and self-esteem are key strategies for reducing caregiver burden, enhancing well-being, and improving the long-term sustainability of care.

## 1. Introduction

Formal and informal caregivers play a vital role in today’s social and healthcare landscape. Their importance is particularly heightened in situations characterized by an aging population, the presence of chronic diseases, and situations of dependency [1]. Performing the tasks associated with caring for a dependent person involves coping with physical, emotional, and cognitive demands that can significantly impact the mental health and quality of life of those who fulfill this role [2]. Traditionally, the scientific literature has focused on analyzing the negative effects of caregiving, highlighting variables such as anxiety, depression, stress, and burnout [3]. However, in recent years, there has been growing interest in studying the positive psychological resources that can act as protective factors against these demands, notably self-efficacy and self-esteem [4].

Self-efficacy, defined as a person’s belief in their ability to organize and carry out the actions necessary to overcome specific situations, is considered a significant predictor of coping strategies in adverse contexts [5]. This variable is related to the perception of control over the caregiver role and promotes more effective adaptation to the demands inherent in caregiving [6]. Furthermore, recent research indicates that self-efficacy can be strengthened through specific interventions aimed at enhancing the caregiver’s internal resources [7].

Self-esteem is another essential psychological resource for fulfilling the role of a caregiver. This construct refers to a person’s overall assessment of themselves and their own personal worth [7]. Compared to self-efficacy, which focuses on the perception of one’s ability to perform specific tasks, self-esteem involves a broader emotional and cognitive assessment of self-concept. High levels of self-esteem are associated with a greater sense of competence, well-being, and personal meaning, while low levels are linked to feelings of guilt, failure, and worthlessness, increasing psychological vulnerability and the risk of developing affective disorders [8,9]. In the context of caregiving, self-esteem is linked to feelings of worth, recognition, and personal satisfaction, allowing caregiving to be viewed not only as a source of burden but also as a potential experience of personal growth when adequate psychological and social resources are available [7].

Furthermore, this construct has a solid theoretical foundation in classical and contemporary psychology. From Rosenberg’s model, which defines it as a person’s overall evaluation of themselves, to Coopersmith’s proposal [10], which emphasizes the role of experiences of success and social recognition, self-esteem emerges as a central resource for adaptation. In the field of caregiving, Leary’s sociometric theory [11] is particularly relevant, as it proposes self-esteem as a “sociometer” that reflects the degree of perceived acceptance or rejection. Caregivers, when facing situations of social isolation, loss of previous roles, or criticism related to their performance, may experience declines in self-esteem that increase their emotional vulnerability. Likewise, from the perspective of Hobfoll’s resource conservation model [12], self-esteem is considered a basic psychological resource that, when strengthened, protects against care-related burnout and cushions the effects of overload. This conceptual framework allows us to understand how self-esteem, beyond being an individual trait, constitutes a dynamic and modulating factor within the caregiving experience, continuously interacting with self-efficacy and other internal resources.

Recent scientific literature has shown clear associations between self-esteem and mental health disorders, demonstrating that low levels of self-esteem are linked to a higher prevalence of anxiety and depression among caregivers [8]. Kim [13] observed that caregivers with low self-esteem exhibited significantly higher levels of stress and depression. Similarly, Yang et al. [14] highlighted the mediating role of this variable in the relationship between the patient’s health status and the caregiver’s perceived satisfaction. When the caregiver’s self-esteem was high, satisfaction remained stable regardless of the patient’s level of dependency; however, when self-esteem was low, satisfaction decreased in parallel with clinical deterioration.

In this vein, self-efficacy and self-esteem should not be understood as independent and isolated dimensions, but rather as interrelated psychological constructs that influence one another in the context of caregiving. Self-efficacy, by strengthening the belief in one’s own ability to tackle tasks and challenges, can contribute to the development of self-esteem, especially in contexts where personal performance is subject to constant evaluation, as is the case in the long-term care of dependent individuals. In turn, strong self-esteem can reinforce perseverance, motivation, and effective coping, thereby increasing the perception of self-efficacy. In this way, both variables form a bidirectional cycle of reinforcement [15].

On the other hand, Wang et al. [16] reported that informal caregivers of people with neurodegenerative diseases who had high levels of self-esteem acted as moderators of the impact of perceived stress on self-efficacy, indicating that higher self-esteem helps maintain confidence in one’s ability to provide care even in highly demanding situations. Conversely, when self-efficacy is low—that is, when the caregiver perceives that they cannot adequately manage caregiving tasks—a decrease in self-esteem is often observed, especially if this perception is accompanied by feelings of guilt or failure.

This interaction has also been observed among formal caregivers, such as social and healthcare technicians or nursing home staff [17]. Molero et al. [15] studied formal caregivers and found that those with higher levels of self-efficacy had significantly higher levels of self-esteem and lower levels of emotional exhaustion. This suggests that the development of skills and the recognition of one’s own capabilities in the workplace directly impact professional self-esteem.

Although recent scientific evidence has shown significant associations between self-efficacy, self-esteem, and various indicators of psychological well-being among caregivers, the available theoretical framework remains heterogeneous and fragmented in terms of methodology, study populations, and outcomes. First, most studies have examined self-efficacy and self-esteem independently, limiting understanding of how these psychological resources may jointly influence caregiver burden and adaptation. Second, research has been conducted across highly diverse caregiving contexts, including disability, dementia, chronic illness, and other dependency conditions, making it difficult to integrate findings across populations. Third, existing studies have focused on a broad range of outcomes, such as stress, depression, resilience, coping, and quality of life, while evidence specifically addressing caregiver burden remains dispersed.

To the best of our knowledge, no recent systematic review has synthesized the evidence on self-efficacy and self-esteem simultaneously while specifically examining their relationship with caregiver burden across both formal and informal caregivers. As a result, the extent to which these psychological resources act as protective factors against caregiver burden remains insufficiently clarified.

Therefore, the present study aims to synthesize the available scientific evidence on the relationship between self-efficacy, self-esteem, and caregiver burden, with the goal of providing an integrative framework that contributes to guiding future research and the design of interventions aimed at caregiver well-being. By integrating findings from different caregiving settings and populations, this review seeks to clarify the protective role of these psychological resources and identify implications for future intervention development and caregiver support strategies.

## 2. Materials and Methods

### 2.1. Design

This study is based on a systematic review of scientific publications focusing on self-efficacy, self-esteem, and overload in formal and informal caregivers of dependent individuals. The aim of this systematic review was to synthesize the available scientific evidence on the relationship between self-efficacy, self-esteem, and caregiver burden among formal and informal caregivers of dependent individuals, as well as to identify their potential implications for caregiver well-being and intervention development. To ensure a rigorous and systematic procedure, the PRISMA methodology [18] was applied, which clearly structures the phases of search, selection, and analysis of the studies (see Table S2 for the PRISMA checklist). Likewise, the review protocol was registered in PRÓSPERO (ID: CRD420251162157/ https://www.crd.york.ac.uk/PROSPERO/view/CRD420251162157 accessed on 15 March 2026), ensuring the transparency and replicability of the study.

The search was conducted in scientific databases such as Scopus, Dialnet, ScienceDirect, Web of Science, and PubMed, using specific equations constructed from the study variables. After identifying the results, previously defined inclusion and exclusion criteria were applied, selecting only those studies that met them. Subsequently, the methodological quality of the selected studies was evaluated using a checklist composed of five basic items, which allowed the information to be organized according to its validity and relevance. Finally, the results were classified into different thematic categories in order to facilitate an accurate analysis aligned with the objectives of the study. This approach provided a clear overview of the current state of the art and the gaps in the literature.

### 2.2. Sources of Information

The literature search focused on specialized databases (Scopus, Dialnet, ScienceDirect, Web of Science, and PubMed) in order to identify a representative number of studies on the topic in question. Studies from different geographic and linguistic contexts were included to provide a more comprehensive and diverse perspective. The screening and selection of studies was conducted independently by two authors using the Convidence platform. Disagreements that arose during the study selection and eligibility phases were resolved through discussion and consensus among the reviewers until a final decision was reached.

The search equation used was constructed based on the three main variables of the study: self-efficacy (1), self-esteem (2), and overload (3), using the Boolean operators AND and OR to optimize the accuracy and relevance of the results. The final formula was: (“self-efficacy”) AND (“self-esteem” OR “self-concept”) AND (caregiver OR carer OR “family caregiver” OR “informal caregiver” OR “formal caregiver”) AND (“caregiver burden” OR “care burden” OR “caregiver strain” OR “caregiver stress” OR “role overload).

To broaden the scope and avoid biases arising from a single combination of descriptors, the main equation was divided into two complementary equations, using the terms “self-efficacy” and “self-esteem” or “self-concept” independently. This methodological decision allowed for a more comprehensive and specific view of the phenomenon, ensuring the thoroughness of the process.

Finally, to clearly define the focus of this review and properly structure the research question, the PICOS strategy was applied [19]. The question guiding this study is: What is the relationship between self-efficacy and self-esteem and caregiver burden in caregivers of dependent individuals? Table 1 shows the relationship between the variables of interest and the components included in the PICOS strategy, specifying the criteria applied to each category.

The variables of self-esteem and self-efficacy were analyzed both independently and jointly, always maintaining their relationship and impact on caregiver burden as the main focus of this literature review. Table 2 shows the equation used in each database, together with the number of results obtained. The search process was carried out during the first half of March 2026.

The same equations were used in all search sources, with the exception of the ScienceDirect and Dialnet databases, where it was necessary to reduce the equation due to the limitations of these platforms in handling broad search fields.

### 2.3. Selection Criteria

To ensure the relevance and quality of the studies included, this review established clearly defined inclusion and exclusion criteria. Table 3 details the criteria applied in each case:

Systematic reviews and meta-analyses were excluded because the aim of the present review was to synthesize evidence derived exclusively from primary empirical studies, thereby avoiding duplication of findings already reported in secondary research. Likewise, studies focused solely on the validation, adaptation, or psychometric evaluation of scales and questionnaires were excluded, as they did not directly examine the relationship between self-efficacy, self-esteem, and caregiver burden, which constituted the primary focus of this review. Furthermore, gray literature, including dissertations, conference proceedings, reports, and other non-peer-reviewed documents, was not considered for inclusion.

## 3. Results

### 3.1. Selection of Studies

**Figure 1:**
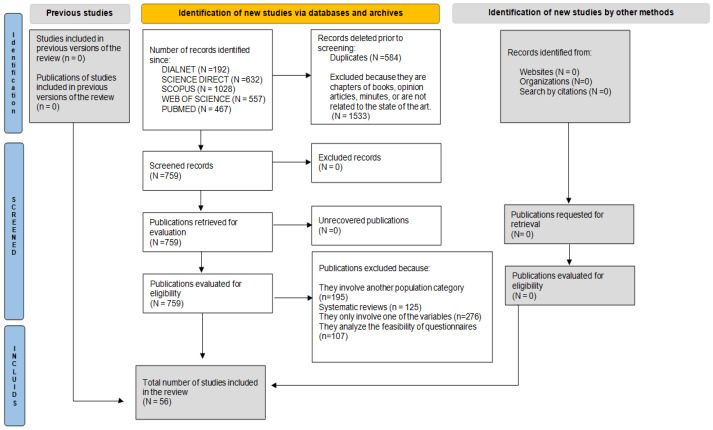
PRISMA diagram.

After searching the different databases, 2876 publications were identified. Of these, 584 were eliminated due to duplication between sources. Subsequently, in the first screening phase, based on title, abstract, and exclusion of protocols, book chapters, opinion articles, or proceedings, 1533 studies were discarded. The 759 articles that passed this stage were evaluated in full text using the previously defined inclusion and exclusion criteria, resulting in a total of 56 studies being selected. The entire selection and evaluation process was carried out systematically and rigorously by the group of authors, following the guidelines established in the registered protocol (Figure 1).

### 3.2. Article Quality Assessment Process

The methodological quality of the studies included in this systematic review was assessed using the Newcastle–Ottawa Scale (NOS) adapted for cross-sectional studies [20], which is derived from the current version of the original scale developed for cohort and case–control studies by Wells et al. [21]. This adapted version incorporates modifications to the evaluation criteria regarding selection, including factors related to representativeness, sample size, and exposure assessment, while maintaining the general structure of the domains: Selection, Comparability, and Outcomes [20]. This adaptation was chosen due to the prevalence of cross-sectional studies among the included studies.

For cross-sectional studies, the maximum score assigned was Selection (5 points), Comparability (2 points), and Outcome (3 points), for a total of 10 points. The overall quality rating was made according to the following guidelines: high quality (HQ) of 8–10 points (low risk of bias), medium quality (MQ) of 6–7 points (medium risk of bias), and low quality (LQ) of less than 5 points (high risk of bias).

The assessment was conducted independently by four reviewers with extensive training and experience in research and pedagogical models. Discrepancies were resolved through discussion until consensus was reached. Table 4 describes the results of the quality assessment of the 56 studies.

After evaluating inter-rater reliability using the intraclass correlation coefficient (ICC), an ICC (2,k) value of 0.963 was obtained with a 95% confidence interval (CI) [0.948–0.975], indicating excellent reliability among raters. The ICC for individual measurements was 0.868 with a 95% CI [0.821–0.908], ensuring solid consistency in the scores assigned by each evaluator independently.

### 3.3. Study Characteristics

Table S1, available in the “Supplementary Materials,” provides a detailed summary of the 56 studies included in this review. The table presents key information about each study, including the authors and year of publication, methodological design, intervention or program implemented (where applicable), sample characteristics, main findings, and reported limitations. Overall, the included studies spanned diverse care settings and populations, providing a comprehensive overview of the relationship between self-efficacy, self-esteem, and caregiver burden among both formal and informal caregivers.

### 3.4. Summary of Results

#### 3.4.1. Methodological Analysis

The studies included can be grouped into three main lines of research. First, articles examining the relationship between self-efficacy and caregiver burden. A negative association is observed: greater self-efficacy is associated with less perceived overload and better mental health [22,29,67]. Second, publications that describe intervention programs aimed at strengthening self-efficacy and self-esteem as a protective resource. Among the interventions identified, there are various designs aimed at strengthening the personal resources of caregivers. These include self-management programs [46], physical activity-based initiatives [41], spiritual support strategies [23], home care [38], and collaborative models of home care and follow-up such as Care Ecosystem [30]. In addition, digital or virtual programs have been created that seek to improve coping skills and promote consistent and continuous support, including CONNECT, OncoKompas, Minds Together, Partner in Balance, Visual Arts, Savvy Advanced, among others [39,43,48,49,50,56,57]. Thirdly, there are studies that explore self-esteem in caregivers, although these are few and far between and, in most cases, appear as a secondary variable linked to self-efficacy or resilience [16,60].

#### 3.4.2. Characteristics of the Population

Studies show a higher prevalence of informal caregivers, primarily immediate family members. Their average age ranges from 45 to 60 years, although the range also extends from young adults (around 20 years old) to people over 70 years old [39,42]. This range reflects the diversity of profiles, with the most common group being caregivers of working age, who balance caregiving with their work responsibilities, thereby increasing the risk of burnout.

Although informal caregivers represented the majority of the included samples, several studies also examined formal caregivers, including healthcare professionals and residential care staff. Informal caregivers were predominantly family members providing long-term unpaid care, whereas formal caregivers were characterized by their professional caregiving responsibilities within healthcare and social care settings. These differences should be considered when interpreting the findings, as the caregiving context, occupational demands, and sources of burden may differ between both groups.

Furthermore, the feminization of the caregiving role is also confirmed, with women accounting for 60–80% of the samples [16,23]. This pattern is consistent with previous literature, which identifies women as the group most vulnerable to the emotional and physical demands associated with caregiving. Beyond its descriptive nature, this finding is relevant because various studies have shown that female caregivers experience higher levels of overload, anxiety, and emotional exhaustion, as well as lower levels of self-efficacy in high-demand contexts.

Gender differences may influence the regulation of internal psychological resources, such as self-esteem and self-efficacy, in response to caregiving-related stress, thereby shaping the coping strategies employed and the subjective perception of personal competence. In this regard, the need to incorporate a gender perspective into future research and interventions is emphasized, with the aim of promoting understanding of how these psychological variables operate differently in male and female caregivers [74].

#### 3.4.3. Quality and Methodological Design

Most articles were classified as high quality (*n* = 37), which lends strength to the findings. A smaller number of studies received a medium quality rating (*n* = 19), mainly due to limited description of key aspects related to the sample, methodology, and acknowledged limitations. Cross-sectional designs predominate, providing significant correlations between self-efficacy, self-esteem, and overload, although they limit the ability to establish causal relationships [22,47,67].

In terms of geographical distribution, there is a clear imbalance in the origin of the selected studies. The region with the highest prevalence of studies is Asia, with 32 articles from China, Turkey, Iran, South Korea, Japan, Indonesia, Malaysia, Israel, Hong Kong, and Taiwan. In Europe, six studies were identified, conducted in Italy, the Netherlands, Poland, Switzerland, and Ireland. In the Americas, 14 studies were found, from the United States, Canada, Mexico, and Cuba. Although the representation of studies from Latin America is limited, with only two studies, Africa also had a low prevalence, with only one study conducted in Nigeria, and in Oceania, one study was recorded from Australia.

This distribution, with a high prevalence in Asia, moderate presence in Europe and North America, and low prevalence in Latin America and Africa, hinders a global cross-cultural analysis of the findings and highlights the need for increased research in less represented sociocultural contexts.

#### 3.4.4. Relationships Observed Between Self-Esteem, Self-Efficacy, and Overload

The 56 studies included in the systematic review showed an association between self-esteem, self-efficacy, caregiver burden, and various indicators of mental health in both formal and informal caregivers. Although these associations were consistently observed across both groups, some differences emerged according to the caregiving context. Among informal caregivers, higher self-efficacy was primarily associated with lower perceived burden and better psychological adjustment in the context of long-term family caregiving. In contrast, studies involving formal caregivers mainly highlighted the protective role of self-efficacy and self-esteem in managing work-related demands, emotional exhaustion, and occupational burden. In general, higher levels of self-efficacy were associated with lower levels of perceived burden, while lower perceptions of personal efficacy were linked to higher levels of stress, anxiety, and depressive symptoms [23,30,68].

Several studies have demonstrated positive outcomes following interventions aimed at strengthening self-efficacy and reducing caregiver burden. These studies included spiritual support programs [24], self-management strategies [47], physical activity [43], home-based follow-up [39], as well as digital and virtual support initiatives [39,48]. Most of these interventions were implemented among informal caregivers, although the available evidence also suggests potential benefits for formal caregivers, supporting the relevance of strengthening psychological resources across different caregiving settings. The reported results included increases in perceived confidence in managing care-related demands, as well as improvements in quality of life and mental health indicators. Along these lines, interventions such as Care Ecosystem showed associations with reductions in burden, particularly during the early stages of implementation.

With regard to self-esteem, fewer studies specifically examined this variable. Some articles demonstrated associations between positive levels of self-esteem and higher levels of resilience and satisfaction in the caregiver-care recipient relationship, even in high-demand caregiving contexts [37]. However, in several studies, this variable was assessed indirectly or as a secondary outcome. The evidence also showed associations between low levels of self-esteem and greater vulnerability and emotional overload [61].

Finally, several studies examined the relationship between external factors, social support, self-efficacy, and emotional overload. Studies such as those by Cao et al. [48] and Leung et al. [55] reported associations between social support, higher levels of self-efficacy, lower emotional overload, and a better quality of life. Similarly, Yang et al. [59] found that social support and self-efficacy are jointly associated with lower levels of anxiety and depression.

#### 3.4.5. Analysis of the Effectiveness of Interventions

In summary, the findings indicate that strengthening internal resources such as self-efficacy and self-esteem constitutes a comprehensive strategy for mitigating the overload associated with caregiving. The interventions analyzed demonstrate the existence of diverse, valid, and effective programs to enhance self-efficacy, ranging from face-to-face modalities to virtual and digital proposals, offering useful guidelines for professional caregiving practice. Table 5 presents the programs identified along with their sample size, effect size, and bias, allowing for a more detailed understanding of the effectiveness of each one.

## 4. Discussion

This study highlights the importance of understanding internal psychological resources such as self-efficacy and self-esteem in the experience of caregivers of dependent individuals. Unlike the scientific tradition focused on the negative impact of caregiving (depression, anxiety, burnout), current evidence suggests that these positive capacities play an essential protective role by shaping how caregivers cope with the strain associated with the role. In this regard, one of the main contributions of this study is the systematic integration of these resources from a comprehensive perspective, identifying consistent patterns across various caregiving contexts that reinforce their role as key protective factors beyond the independent approaches previously taken in the scientific literature [74].

Several studies show a clear negative association between self-efficacy and caregiver burden; caregivers who have greater confidence in their abilities not only experience less burden but also report better mental health, fewer symptoms associated with depression and anxiety, and a more positive assessment of their quality of life [23,30,68]. In this vein, the scientific literature demonstrates that self-efficacy functions as a mediating variable in various care contexts, ranging from dementia to cancer, where it has been shown to strengthen coping skills, even in situations where demands were highest [14,74]. This reflects that self-efficacy should not be interpreted solely as a personal variable but as a modifiable competence that can be developed and trained, which opens up a broad field of research aimed at designing novel clinical and community interventions to strengthen it [73]. Based on this synthesis, it is also observed that this pattern persists regardless of the type of caregiver (formal or informal) and the clinical context, which supports the robustness of the finding and its cross-cutting applicability. Furthermore, the review allows us to identify that self-efficacy not only acts as an independent variable but also as a modulating component of more complex adaptation processes during caregiving tasks.

Various projects aimed at strengthening self-efficacy have shown some degree of effectiveness in boosting caregivers’ confidence in managing adverse situations [31,39,42,47]. In general, these interventions have a positive influence on physical and psychological health, as well as on quality of life, reducing perceived burden. Recent studies identify positive effects that extend to other areas such as improved sleep, resilience, and life satisfaction, helping to establish self-efficacy as a stable protective factor [14]. In this regard, mindfulness-based practices and cognitive-behavioral therapy stand out among the most effective strategies for reducing burden and depressive symptoms, particularly among caregivers of people with dementia and chronic illnesses, by reducing anxiety and stress through attention training, emotional regulation, and acceptance. However, this systematic review also reveals significant heterogeneity in the design, duration, and content of the programs, which complicates direct comparison of their effects and underscores the need for future research with more standardized protocols to allow for a more precise evaluation of their efficacy.

As for self-esteem, although it has been studied less specifically, it also emerges as a key protective factor during the adjustment to the role of caregiver. The evidence gathered shows that high self-esteem is associated with greater resilience, satisfaction, and personal competence, which helps mitigate the negative impacts of caregiving [9,36]. Conversely, low levels of self-esteem increase emotional vulnerability and are associated with feelings of guilt, failure, and worthlessness—factors that contribute to the development of depressive and anxiety disorders [8]. It is worth noting that, like self-efficacy, self-esteem also acts as a mediating variable between caregiver burden and quality of life, establishing connections between external demands and internal resources [8]. However, the scarcity of research focused on self-esteem constitutes a significant limitation in this field.

In this regard, one of the most significant findings of this study is the identification of the interaction between self-efficacy and self-esteem as an interdependent psychological system, in which both variables mutually reinforce one another and jointly contribute to adaptation to caregiving—a factor that has rarely been addressed in an integrated manner in the previous literature [67]. Another key variable is social support, which enhances self-efficacy, reduces caregiver burden, and fosters a more positive perception of the caregiver’s role [47,53]. Internal resources (self-efficacy, self-esteem, resilience, etc.) do not operate in isolation but rather interact dynamically with other external resources (social support), which mitigates the effects of burden and contributes to the well-being of both the caregiver and the care recipient. This reinforces an ecological perspective, as it shows that the best outcomes are observed when individual resources are combined with appropriate supportive contexts, suggesting the need for multi-component interventions that integrate personal and social dimensions [68].

Finally, the feminization of care is a topic of interest in recent research, driven by the high proportion of female caregivers. This gender bias increases the burden and limits opportunities for work–life balance [22,35], reinforcing the need for policies that acknowledge the unequal distribution of care and promote support measures in the social and family spheres. Furthermore, this review highlights that gender differences affect not only the distribution of care but also the subjective experience, opening new lines of research aimed at understanding how psychological resources operate differently in male and female caregivers.

### 4.1. Limitations

Despite the reliability and relevance of the results, this review has several limitations. First, although the number of included studies was relatively high, a meta-analysis was not conducted due to the considerable clinical and methodological heterogeneity among the studies. Specifically, considerable differences were identified in the caregiver populations (formal and informal caregivers in various situations of dependency), study designs, outcome measures, and the wide variety of instruments used to assess self-efficacy, self-esteem, and caregiver burden. This level of heterogeneity made a pooled quantitative analysis inappropriate, as combining such diverse data could have yielded misleading summary estimates and reduced the validity of the general conclusions. Therefore, the evidence presented should be considered correlational rather than causal, since most of the included studies are based on designs that preclude inferring cause-and-effect relationships.

Another relevant limitation concerns geographical and population heterogeneity. There has been a greater representation of studies conducted in Asian countries (China, Turkey, Iran, Korea), while research from the United States, Latin America, and Europe is less numerous. This distribution may affect the generalization of the results, considering the sociodemographic and cultural differences that influence self-efficacy, self-esteem, and overload. Likewise, the scarce presence of formal caregivers and the overrepresentation of informal caregivers constitute another bias that limits the applicability of the findings. Additionally, although the search strategy included several major multidisciplinary databases, it did not incorporate databases specifically focused on psychology and nursing, such as PsycINFO, CINAHL, or Embase. Consequently, some potentially relevant studies may not have been identified despite the comprehensive search strategy employed.

These gaps suggest the need for more longitudinal and multicenter research with diverse samples that include both categories of caregivers and different sociocultural contexts. Exploring how self-efficacy and self-esteem evolve over time and in different settings would allow for the generation of more comprehensive and robust evidence.

### 4.2. Implications for Professional Practice and Policy

The results of this review highlight the need to promote self-efficacy and self-esteem as fundamental pillars in public policies supporting caregivers. From an institutional perspective, it is recommended to promote programs aimed at developing self-management skills, psychoeducational training, problem-solving training, and digital support, given their positive association with strengthening personal psychological resources and reducing overload.

In the healthcare field, these variables should be incorporated into the design of multidisciplinary intervention programs, including the active participation of psychologists and educators in care. From a political and social perspective, it is essential to establish monitoring systems that allow for the evaluation of the caregiver’s well-being, considering indicators such as self-efficacy, self-esteem, burden, and quality of life. The implementation of this continuous assessment system would facilitate evidence-based decision-making, strengthening the sustainability and effectiveness of programs.

Finally, it is important to promote training and education programs aimed at professionals in the social and healthcare fields, with the aim of developing skills for the early detection of overload and the promotion of positive psychological resources in caregivers.

## 5. Conclusions

The literature review shows that self-esteem and self-efficacy are directly and consistently associated with the caregiving experience, constituting two fundamental psychological resources for preventing overload and other mental health disorders linked to the role of caregiver. Evidence shows that high levels of self-efficacy are related to a lower perception of burden and the use of more effective and adaptive strategies in response to the physical, emotional, and social demands of caregiving. In the case of self-esteem, although the findings are more limited, the available studies indicate that a positive personal assessment is associated with greater resilience and well-being.

Both variables interact dynamically and complementarily: self-efficacy boosts confidence in one’s own abilities, while self-esteem promotes a positive self-image, generating a combined effect that facilitates more effective coping strategies in the face of caregiving challenges. Understanding this relationship is key to guiding future interventions aimed at reducing caregiver burden, especially in high-demand care contexts.

From a professional and social perspective, it is considered necessary for the institutions and systems responsible for care to introduce an approach focused on the strengths of the caregiver, promoting training and support programs that foster the development of self-efficacy and self-esteem. Along these lines, social and family support should be considered another complementary resource that enhances internal resources and contributes to the overall well-being of the caregiver.

The increase in the dependent population and the persistent feminization of caregiving require policies that recognize the work of caregivers, reduce gender inequalities, and guarantee accessible and effective resources. In this context, promoting self-efficacy and self-esteem should be considered a priority strategy in public and health policies aimed at improving the quality, equity, and sustainability of care.

Finally, although the available evidence is mostly correlational and not causal, the findings suggest that promoting self-efficacy and self-esteem in caregivers is a promising approach to reducing perceived overload and strengthening overall well-being. Integrating these resources into intervention programs and public policies represents an essential step toward building a society that values, respects, and protects the health of those who continuously sustain the invisible network of common well-being.

## Figures and Tables

**Table 1:** *PICOS strategy* *descriptors.*

Components	Description
P	Population: Formal and informal caregivers of dependent persons
I	Constructs of Interest: Self-efficacy and Self-esteem are the psychological variables to be analyzed jointly and individually.
C	Comparison: As no comparative analysis between populations or variables is performed, this term is omitted.
O	Results/Outcomes: Overload, analyzed as the primary outcome, based on the impact that caregiver self-efficacy and self-esteem have on it.
S	Designs: Empirical, cross-sectional, longitudinal, quasi-experimental, clinical trials

**Table 2:** *Search* *equations.*

*Search Equations*	*Database*	*Filters*	*Results*
“self-efficacy” AND “self-esteem” AND caregiver AND “caregiver burden”	Dialnet	0	61
“self-efficacy” AND caregiver AND “caregiver burden”	Dialnet	0	68
“self-esteem” AND caregiver AND “caregiver burden”	Dialnet	0	63
(“self-efficacy”[Title/Abstract] OR “self efficacy”[Title/Abstract]) AND (“self-esteem”[Title/Abstract] OR “self esteem”[Title/Abstract] OR “self-concept”[Title/Abstract] OR “self concept”[Title/Abstract]) AND (caregiver[Title/Abstract] OR carer[Title/Abstract] OR “family caregiver”[Title/Abstract] OR “informal caregiver”[Title/Abstract] OR “formal caregiver”[Title/Abstract]) AND (“caregiver burden”[Title/Abstract] OR “care burden”[Title/Abstract] OR “caregiver strain”[Title/Abstract] OR “caregiver stress”[Title/Abstract] OR “role overload”[Title/Abstract])	PubMed	0	6
(“self-efficacy”[Title/Abstract] AND (caregiver[Title/Abstract] OR carer[Title/Abstract] OR “family caregiver”[Title/Abstract] OR “informal caregiver”[Title/Abstract] OR “formal caregiver”[Title/Abstract]) AND (“caregiver burden”[Title/Abstract] OR “care burden”[Title/Abstract] OR “caregiver strain”[Title/Abstract] OR “caregiver stress”[Title/Abstract] OR “role overload”[Title/Abstract])	PubMed	0	366
(“self-esteem”[Title/Abstract] OR “self-concept”[Title/Abstract] OR “self concept”[Title/Abstract]) AND (caregiver[Title/Abstract] OR carer[Title/Abstract] OR “family caregiver”[Title/Abstract] OR “informal caregiver”[Title/Abstract] OR “formal caregiver”[Title/Abstract]) AND (“caregiver burden”[Title/Abstract] OR “care burden”[Title/Abstract] OR “caregiver strain”[Title/Abstract] OR “caregiver stress”[Title/Abstract] OR “role overload”[Title/Abstract])	PubMed	0	95
TITLE-ABS-KEY ((“self-efficacy”) AND (“self-esteem” OR “self-concept”) AND (caregiver OR carer OR “family caregiver” OR “informal caregiver” OR “formal caregiver”) AND (“caregiver burden” OR “care burden” OR “caregiver strain” OR “caregiver stress” OR “role overload”))	Scopus	0	368
TITLE-ABS-KEY ((“self-efficacy”) AND (caregiver OR carer OR “family caregiver” OR “informal caregiver” OR “formal caregiver”) AND (“caregiver burden” OR “care burden” OR “caregiver strain” OR “caregiver stress” OR “role overload”))	Scopus	Document type: Article. Subject areas: Psychology, Nursing, Neuroscience, Social Sciences	281
TITLE-ABS-KEY ((“self-esteem” OR “self-concept”) AND (caregiver OR carer OR “family caregiver” OR “informal caregiver” OR “formal caregiver”) AND (“caregiver burden” OR “care burden” OR “caregiver strain” OR “caregiver stress” OR “role overload”))	Scopus	Document type: Article. Subject areas: Psychology, Nursing, Neuroscience, Social Sciences	379
TS=(“self-efficacy”) AND TS=(“self-esteem” OR “self-concept”) AND TS=(caregiver OR carer OR “family caregiver” OR “informal caregiver” OR “formal caregiver”) AND TS=(“caregiver burden” OR “care burden” OR “caregiver strain” OR “caregiver stress” OR “role overload”)	Web of Science	0	107
TS=(“self-efficacy”) AND TS=(caregiver OR carer OR “family caregiver” OR “informal caregiver” OR “formal caregiver”) AND TS=(“caregiver burden” OR “care burden” OR “caregiver strain” OR “caregiver stress” OR “role overload”)	Web of Science	Filter: Open access	348
TS=(“self-esteem” OR “self-concept”) AND TS=(caregiver OR carer OR “family caregiver” OR “informal caregiver” OR “formal caregiver”) AND TS=(“caregiver burden” OR “care burden” OR “caregiver strain” OR “caregiver stress” OR “role overload”)	Web of Science	Filter: Open access	102
“self-efficacy” AND “self-esteem” AND caregiver AND “caregiver burden”	Science Direct	0	274
“self-efficacy” AND caregiver AND “caregiver burden”	Science Direct	Filters: Open Access; Research Articles	310
“self-esteem” AND caregiver AND “caregiver burden”	Science Direct	Filters: Open Access; Research Articles	48

**Table 3:** *Inclusion and* *exclusion criteria.*

*Inclusion Criteria*	*Exclusion Criteria*
Original empirical studies that examine the relationship between the variables under study	Systematic reviews, meta-analyses; only original empirical articles are considered.
Interventions targeting formal and informal caregivers of dependent individuals	Studies focusing on patients or other populations who do not serve as caregivers.
Sources in Spanish and English	Studies whose primary objective is the validation, adaptation, or reliability analysis of instruments, without examining their relationship to caregiver burden.
Studies published up to and including March 2026	Articles that address variables without the required relationship
Studies that include interventions designed to build self-esteem and self-efficacy in caregivers, assessing their impact on caregiver burnout	Those derived from the inclusion criteria themselves

**Table 4:** *Quality assessment* *of articles.*

Study	Selection	Comparability	Results	Total	Quality	Bias
Saretta et al. (2026) [21]	2.5	2	2	6.5	MQ	Moderate
Akobundu et al. (2025) [22]	1.5	2	2.5	6	MQ	Moderate
Bagheri et al. (2025) [23]	2.5	2	3	7.5	MQ	Moderate
Ham et al. (2025) [24]	5	2	3	10	HQ	Low
Kocabas et al. (2025) [25]	4	2	3	9	HQ	Low
Lafuenti et al. (2025) [26]	4.5	2	3	9.5	HQ	Low
Mundakir et al. (2025) [27]	4	0.5	3	7.5	MQ	Moderate
Noguchi et al. (2025) [28]	4	2	2.5	8.5	HQ	Low
Par and Çakir (2025) [29]	4	2	3	9	HQ	Low
Possin et al. (2025) [30]	5	1.5	2	8.5	HQ	Low
Soltys (2025) [31]	2.5	1.5	2.5	6.5	MQ	Moderate
Tez and Ciydem (2025) [32]	4	1	3	8	HQ	Low
Nasreen et al. (2024) [33]	5	2	2.5	9.5	HQ	Low
Özdemir and Elmaoğlu (2024) [34]	4	1	2.5	7.5	MQ	Moderate
Wang et al. (2024a) [16]	4	1	2	7	MQ	Moderate
Wang et al. (2024b) [35]	3.5	1.5	2	7	MQ	Moderate
Wang et al. (2024c) [36]	3.5	1.5	2.5	7.5	MQ	Moderate
Zhang et al. (2024) [37]	3.5	1.5	2	7	MQ	Moderate
Ay and Koç (2023) [38]	4.5	1.5	2	8	HQ	Low
Fitzgeraldson et al. (2023) [39]	3	0.5	2.5	6	MQ	Moderate
Gursoy et al. (2023) [40]	4	1.5	2	7.5	HQ	Low
Kim et al. (2023) [41]	4	0.5	2.5	7	MQ	Moderate
Kim and Park (2023) [42]	4.5	1.5	3	9	HQ	Low
Semere et al. (2023) [43]	4.5	1.5	3	9	HQ	Low
Sun et al. (2023) [44]	4	1.5	2.5	8	HQ	Moderate
Yang et al. (2023) [45]	3.5	1.5	2	7	MQ	Moderate
Banitalebi et al. (2022) [46]	4	1.5	2.5	8	HQ	Low
Cao et al. (2022) [47]	5	2	2.5	9.5	HQ	Low
Cheng et al. (2022) [8]	5	2	3	10	HQ	Low
Noel et al. (2022) [48]	4	1.5	2	7.5	MQ	Moderate
Schuit et al. (2022) [49]	5	2	3	10	HQ	Low
Yang et al. (2022) [14]	3.5	1	3	7.5	MQ	Moderate
Bruinsma et al. (2021) [50]	2.5	1.5	2	6	MQ	Moderate
Rudelli et al. (2021) [51]	4	1.5	2	7.5	MQ	Moderate
Ben–Pazi et al. (2020) [52]	4	1.5	2,5	8	HQ	Low
Leung et al. (2020) [53]	4	1.5	3	8.5	HQ	Low
Rabiei et al. (2020) [54]	5	2	2.5	9.5	HQ	Low
Ramzani et al. (2019) [55]	4	0.5	2	6.5	MQ	Moderate
Richards et al. (2019) [56]	5	2	3	10	HQ	Low
Samia et al. (2019) [57]	5	1	2	8	HQ	Low
Yang et al. (2019) [58]	4.5	1	2	7.5	MQ	Moderate
Boyacıoğlu and Kutlu (2017) [59]	4.5	1.5	3	9	HQ	Low
Gonzalez-Guerra et al. (2017) [60]	2,5	1	2,5	6	MQ	Moderate
MacDougall and Steffen (2017) [61]	4.5	1.5	2.5	9.5	HQ	Low
Yildiz et al. (2017) [62]	3.5	1	2	7.5	MQ	Moderate
Duggleby et al. (2016) [63]	5	2	3	10	HQ	Low
Pablo-Santiago et al. (2016) [64]	3.5	1	2	6.5	MQ	Moderate
Univer et al. (2016) [65]	4.5	1	2.5	8	HQ	Low
Weiss et al. (2016) [66]	5	1	3	9	HQ	Low
Jennings et al. (2015) [67]	3.5	1.5	3	8	MQ	Moderate
Durmaz and Okanli (2014) [68]	3.5	1	2	6.5	MQ	Moderate
Shankar et al. (2014) [69]	5	1.5	3	9.5	HQ	Low
Zhang et al. (2014) [70]	4	1	3	8	HQ	Low
Gallagher et al. (2011) [71]	3.5	1.5	3	8	HQ	Low
Merluzzi et al. (2011) [72]	4	1	3	8	HQ	Low
Gonyea et al. (2005) [73]	4	1	2.5	7.5	MQ	Moderate

Note: HQ: high quality. MQ: medium quality.

**Table 5:** *Intervention* *programs.*

Authors	Design	Sample	Program	Effect Size	Bias
Saretta et al. (2026) [21]	Quasi-experimental pilot study	103 caregivers	Savvy Caregiver	A significant negative correlation was found between self-efficacy and caregiver burden (r = −0.34, *p* < 0.01).	Moderate
Bagheri et al. (2025) [23]	Randomized clinical trial	64 caregivers (32 intervention group and 32 control group)	Spiritual care program. Eight weeks of 30–45 min sessions.	Significant increases in self-efficacy (*p* < 0.01) and reductions in overload levels (*p* < 0.05) were observed in the intervention group compared to the control group.	Moderate
Possin et al. (2025) [30]	Randomized clinical trial	456 caregivers	Collaborative dementia care program: Care Ecosystem	Participation was associated with greater self-efficacy (*p* < 0.05) and lower caregiver depression (*p* < 0.05) and a tendency toward lower overload (*p* = 0.07).	Moderate
Ay and Koç (2023) [38]	Quasi-experimental pretest-posttest design	40 caregivers (20 experimental group and 20 control group)	Program: Home care and follow-up. Six sessions lasting 60 to 90 min.	Mothers showed less state anxiety, trait anxiety, and caregiver burden. In addition, there was a significant increase in parental self-efficacy (all with *p* < 0.0001) compared to the control group.	Moderate
Kim et al. (2023) [41]	Quasi-experimental pretest-posttest design	64 caregivers (34 experimental group and 30 control group)	Program: Physical Activity. Eight weeks and 60 min per week	The experimental group showed improvements in self-efficacy for self-care (*p* < 0.001), exercise (*p* < 0.001), physical function (*p* < 0.001), and health-related quality of life (*p* < 0.001). In addition, lower levels of caregiver burden (*p* < 0.001) and depressive symptoms (*p* < 0.001) were observed.	Moderate
Semere et al. (2023) [43]	Randomized clinical trial	381 caregivers	Program: CONNECT	No significant differences were observed for stress, depression, or anxiety, but self-efficacy showed a significant increase.	Moderate
Banitalebi et al. (2022) [46]	Quasi-experimental design	70 caregivers (35 experimental group and 35 control group)	Program: Self-management. Eight sessions.	After the intervention, there was a decrease in the burden of care (mean intervention = 42.6 ± 3; at 3 months = 36 ± 3; *p* < 0.001) and an increase in the level of self-efficacy (mean immediate intervention = 60.6 ± 4.7; at 3 months = 72.7 ± 4; *p* < 0.001).	Moderate
Noel et al. (2022) [48]	Quasi-experimental design with pretest and posttest	134 caregivers (90 in the experimental group and 44 in the control group)	Program: Virtual education. Five synchronous online training sessions.	Improvements were observed in the caregiver’s level of confidence and self-efficacy (*p* < 0.01). However, no significant differences were found in caregiver burden between the two groups.	Moderate
Schuit et al. (2022) [49]	Randomized clinical trial	58 caregivers (28 experimental group and 30 control group)	Program: OncoKompas	No significant differences were observed in caregiver burden, self-efficacy, or health-related quality of life (HRQOL) when comparing the intervention and control groups at 3 months.	Moderate
Bruinsma et al. (2021) [50]	Quasi-experimental design with pretest and posttest	20 caregivers	Program: Partner in Balance	Significant improvements in self-efficacy were identified, and symptoms of anxiety and depression were reduced following the intervention (*p* ≤ 0.05).	High
Rabiei et al. (2020) [54]	Randomized clinical trial	70 caregivers (35 experimental group and 35 control group)	Family-Centre Red Intervention/Empowerment Program.	Significant improvements in self-efficacy (*p* < 0.001) and a significant reduction in caregiver burden (*p* < 0.01) were identified in the intervention group compared to the control group.	Moderate
Richards et al. (2019) [56]	Randomized clinical trial	26 caregivers (14 in the experimental group and 12 in the control group)	Program: Visual arts. One session per week for two months.	There was a significant increase in participants’ self-esteem (*p* < 0.05) and a significant reduction in burden (*p* < 0.05) compared to the control group.	Low
Samia et al. (2019) [57]	Quasi-experimental study	100 caregivers	Program: Savvy Advanced	Significant improvements were identified between baseline and after 5 months of intervention in competence (*p* = 0.023), personal gain (*p* = 0.002), self-efficacy (*p* = 0.038), and depressive symptoms (*p* = 0.005). At 12 months, improvements were maintained in competition (*p* = 0.026) and personal gain (*p* = 0.002).	Moderate

## Data Availability

No new data were created or analyzed in this study. Data sharing is not applicable to this article.

## Supplementary Materials

The following supporting information can be downloaded at https://www.mdpi.com/article/10.3390/healthcare14152267/s1, Table S1. Summary results. Table S2. PRISMA Checklist.

## Abbreviations

The following abbreviations are used in this manuscript:

AFP	Authoritarian filial piety
ASD	Autism Spectrum Disorder
CI	Confidence interval
HQ	High quality
ICC	Intraclass Correlation Coefficient
LQ	Low quality
MQ	Medium quality
N	Number
NOS	Newcastle–Ottawa Scale
NR	Not reported
PRISMA	Preferred Reporting Items for Systematic Reviews and Meta-Analyses.
PROSPERO	International Prospective Register of Systematic Reviews
