# Supplementary material for: Impact of Self-Efficacy and Self-Esteem on Caregiver Burden in Formal and Informal Caregivers of People with Dependency: A Systematic Review
Source: Healthcare (Basel). 2026 Jul 24;14(15):2267. doi: 10.3390/healthcare14152267 (PMC13464599; doi:10.3390/healthcare14152267)
Supplement: Supplementary file 1 [file healthcare-14-02267-s001.zip › healthcare-4228903-supplementary.pdf]

Table S1. Summary results.

Author/Year	Objectives	Methodological Design/Program	Sample	Results	Limitations
Saretta et al., (2026) [21]	To evaluate the effect of the Savvy Caregiver Program on the self-efficacy of caregivers of people with intellectual disabilities.	Quasi-experimental pilot study<br>Program: Savvy Caregiver	n= 103 caregivers<br>Category: Informal<br>Age: 65.48 years<br>Gender: 26.9% men and 73.1% women<br>Region: Italy	Following the intervention, a significant improvement in self-efficacy was observed. This self-efficacy showed negative correlations with burden and positive correlations with social support. Furthermore, the program also improved problem-solving coping.	Non-randomized design, so selection bias may exist.
Akobundu et al. (2025) [22]	To evaluate the relationship between the degree of self-efficacy, overload, and social support	Cross-sectional design	n = 80 caregivers<br>Category: Informal<br>Age: 37.9 years<br>Gender: 25% men and 75% women<br>Region: Nigeria (Africa)	Greater self-efficacy was associated with lower burden, while low self-efficacy was related to higher caregiving burden. In addition, perceived social support acted as a protective factor, encouraging a reduction in burden among caregivers with higher self-efficacy.	Participants were informal caregivers from clinics offering free, specialized care, which may not reflect the experiences of caregivers who do not attend such clinics.
Bagheri et al. (2025) [23]	To analyze the relationship between self-efficacy and overload and the impact of a spiritual care program	Cross-sectional design<br>Program: Spiritual Care	n = 64 caregivers<br>Category: Informal<br>Age: NR<br>Gender: Women<br>Region: Iran (Asia)	The spiritual program produced a significant increase in self-efficacy and a significant reduction in overload, establishing a negative correlation between the two. This situation influences the adequacy of child care, since stress, tension, hospitalization, or family involvement can worsen it.	The small sample size may limit the generalizability of the results
Ham et al. (2025) [24]	To analyze the association between the presence of	Cross-sectional design	n = 1,419 caregivers<br>Category: Informal<br>Age: NR<br>Gender: NR	Caregivers of older adults with multiple chronic diseases experienced greater burden. On the other hand, self-efficacy and self-esteem acted as protective factors	NR

	chronic diseases and perceived overload		Region: United States (America)	that mitigated the effect of multimorbidity on the burden of care.	
Kocabaş et al. (2025) [25]	To analyze the effect of self-efficacy together with resilience on overload	Descriptive correlational cross-sectional design	n = 105 caregivers<br>Category: Informal<br>Age: 57.2 years<br>Gender: 38.1% men and 61.9% women<br>Region: Turkey (Asia)	A higher level of self-efficacy was associated with a lower degree of overload.Caregiver levels of respite, psychological resilience, and self-efficacy to control disturbing thoughts about caregiving significantly predicted caregiver burden.	As this is a cross-sectional design, it may be difficult to establish causal relationships.
Lafuenti et al., (2025) [26]	To explore the relationship between resilience, self-efficacy, and psychological burden in caregivers	Cross-sectional observational design	n = 123 caregivers<br>Category: Informal<br>Age: NR<br>Gender: NR<br>Region: Italy (Europe)	Resilience acted as a mediating tool in the relationship between self-efficacy and caregiver burden. Greater self-efficacy is associated with greater resilience, which reduces psychological burden.	When intervening in a specific palliative care context in Italy, generalization is limited.
Mundakir et al. (2025) [27]	To examine the mediating role of self-efficacy in the relationship between caregiving burden and parental stress	Cross-sectional design	n = 204 caregivers<br>Category: Informal<br>Age: 45.6 years<br>Gender: 32.4% men and 67.6% women<br>Region: Indonesia (Asia)	Parental self-efficacy acted as a mediating resource because higher levels of self-efficacy reduced the impact of the burden on stress. The burden of caregiving was positively associated with parental stress.	When presenting a cross-sectional design, there may be difficulties in establishing causal relationships.
Noguchi et al. (2025) [28]	To examine how learning experiences in caregiving knowledge and skills influence	Cross-sectional design	n = 934 caregivers<br>Category: Informal<br>Age: 58.4 years<br>Gender: NR<br>Region: Japan (Asia)	Caregivers with greater exposure to learning experiences where they acquired caregiving knowledge and skills had higher self-esteem and lower perceived burden. The acquisition of skills was	When presenting a cross-sectional design, it is difficult to establish causal relationships.

	overload and self-esteem			shown to be a protective factor in the caregiving experience.	
Pars & Çakir (2025) [29]	To examine the interrelationship between caregiver overload, parental stress, and self-efficacy	Descriptive correlational cross-sectional design	n = 139 caregivers<br>Category: Informal<br>Age: 37.2 years<br>Gender: Women<br>Region: Turkey (Asia)	A negative correlation was found between coping self-efficacy and caregiver burden. Greater self-efficacy was associated with lower burden. The same was true for stress levels. Lack of training among caregivers and poor social support emerged as variables that increase burden.	The findings are based on statements made by families, so there may be limitations in terms of recall bias.
Possin et al. (2025) [30]	To evaluate the long-term effects of the Care Ecosystem collaborative model on caregivers' self-efficacy and burden.	Pragmatic randomized clinical design<br>Program: Care Ecosystem	n = 456 caregivers<br>Category: Informal<br>Age: 63 years<br>Gender: 25% men and 75% women<br>Region: NR	There was an improvement in caregiver self-efficacy and a reduction in depressive symptoms, along with overload. The effects were more pronounced in the first two years of implementation, reinforcing the importance of collaborative and structured interventions to strengthen self-efficacy as a protective resource against the burden of care.	NR
Sołtys, (2025) [31]	To evaluate caregivers' life satisfaction and associated factors	Cross-sectional design	n = 100 caregivers<br>Category: Informal<br>Age: NR<br>Gender: 22% men and 78% women.<br>Region: Poland (Europe)	Greater perceived self-efficacy and positive role appraisal were associated with greater life satisfaction. This life satisfaction showed a negative correlation with burden; the greater the satisfaction, the more the burden and symptoms of depression were reduced.	The sample was small and located only in Poland, which limits the generalizability.
Tez & Ciydem (2025) [32]	To analyze the relationship between self-stigmatization and caregiver burden	Cross-sectional design	n = 399 caregivers<br>Category: Informal<br>Age: NR<br>Gender: Women<br>Region: Turkey (Asia)	Caregivers with higher self-efficacy and self-esteem reported less stigma and burden, highlighting the protective role of these psychological resources. Self-stigmatization was positively associated with burden; the greater the stigma, the greater the burden.	The sample is limited to caregivers of children with chronic illness, reducing generalizability.

Nasreen et al. (2024) [33]	To describe the burden on caregivers, mental health, quality of life, and self-efficacy of caregivers.	Cross-sectional design	n = 121 caregivers<br>Category: Informal<br>Age: NR<br>Gender: NR<br>Region: Malaysia (Asia)	Caregiver self-efficacy is positively related to social support and the patient's ability to self-care. Caregivers with higher self-efficacy report better quality of life and lower perceived burden.	When presenting a cross-sectional design, there may be difficulties in establishing causal links.
Özdemir & Elmaoğlu (2024) [34]	To analyze the relationship between self-efficacy, care burden, and hopelessness	Cross-sectional design	n = 164 caregivers<br>Category: Informal<br>Age: 32.82 years<br>Gender: Women<br>Region: Turkey (Asia)	Self-efficacy showed a negative correlation with the burden of care and hopelessness. The greater the self-efficacy, the lower the perceived burden and the lower the level of hopelessness. The burden of care is positively associated with hopelessness, and self-efficacy acts as a protective mechanism.	The sample consists only of mothers, which makes generalization difficult.
Wang et al. (2024a) [16]	To analyze the factors associated with overload	Multicenter longitudinal design	n = 185 caregivers<br>Category: Informal<br>Age: 45.3 years<br>Gender: 48.6% men and 51.4% women<br>Region: China (Asia)	Self-efficacy acted as a protective factor against overload. Good self-efficacy was associated with low levels of burden and vice versa. Healthcare personnel can support family caregivers through education, accompaniment, and setting positive examples, thereby helping to strengthen their self-efficacy.	The participants who were selected belong to tertiary hospitals in different regions.
Wang et al. (2024b) [35]	To analyze the trajectory of overload and associated factors in family caregivers of people with colorectal cancer during treatment	Multicenter observational longitudinal design	n = 185 caregivers<br>Category: Informal<br>Age: 45 years<br>Gender: 49% men and 51% women<br>Region: China (Asia)	The burden increased during the middle phase of chemotherapy and subsequently decreased. Self-efficacy and the family member's caregiving capacity were identified as the most decisive factors, along with social support, significantly influencing the overall burden and its specific trajectories.	The selected hospitals were tertiary hospitals from different regions, so the influencing factors explored only apply to primary care hospitals under the same conditions.

Wang et al. (2024c) [36]	To examine the impact of internal and external resources on family overload and psychological distress.	Cross-sectional design	n = 195 caregivers<br>Category: Informal<br>Age: 55.81 years<br>Gender: 34.20% men and 65.80% women<br>Region: China (Asia)	Family overload was associated with greater psychological distress, while self-efficacy and positive coping acted as protective internal resources, significantly mediating this relationship. Caregivers with greater self-efficacy experienced less impact from overload on their mental health, highlighting the importance of strengthening confidence in one's own abilities to reduce the negative effects of caregiving.	The cross-sectional design makes it more difficult to establish causal relationships.
Zhang et al., (2024) [37]	To explore the role of self-efficacy and authoritarian filial piety (AFP) in caregiver burden.	Cross-sectional design	n = 601 caregivers<br>Category: Informal<br>Age: 53.04 years<br>Gender: 38% men and 62% women<br>Region: China (Asia)	Self-efficacy mediated the relationship between the burden and benefits of caregiving. AFP moderated the relationships between burden and self-efficacy and burden and benefits, such that higher AFP can weaken the positive effects of self-efficacy.	Information was collected from a specific Chinese context, which limits generalizability.
Ay & Koç (2023) [38]	To evaluate the effect of home care and follow-up on mothers who are caregivers	Quasi-experimental design, pre-test/post-test<br>Program: Home care and follow-up	n = 40 caregivers<br>Category: Informal<br>Age: 21-40 years<br>Gender: Women<br>Region: Turkey (Asia)	The intervention resulted in a significant increase in self-efficacy and a marked decrease in overload. These findings show that structured support can: strengthen mothers' confidence in their caregiving abilities, reduce emotional burden, and improve caregiving practices.	Care needs may vary from one mother to another. This could cause difficulties when generalizing the results.
Fitzgeraldson et al. (2023) [39]	To analyze the influence of a program on the mental health of caregivers	Mixed design<br>Program: Minds together	n = 108 caregivers<br>Category: Informal<br>Age: 50 years<br>Gender: 11% men and 91% women<br>Region: Australia (Europe)	The program that caregivers underwent increased their self-efficacy in coping during caregiving, which led to a reduction in caregiver burden. Self-esteem was not analyzed independently, but the findings suggest that digital interventions	The sample collected consisted of caregivers with depressive or anxiety disorders, which makes it difficult to generalize the results.

				may be a way to strengthen psychological resources in caregivers	
Gursoy et al. (2023) [40]	To analyze the association between the progression of children's disease and depression, burnout, burden, and self-esteem in their caregivers.	Cross-sectional design	n = 85 caregivers<br>Category: Informal<br>Age: NR<br>Gender: Women<br>Region: Turkey (Asia)	Negative child development was associated with higher levels of depression, burnout, and overload in mothers. Self-esteem was identified as a protective psychological mechanism, cushioning the negative effects of caregiving.	The study sample was limited to mothers, excluding fathers and other family members.
Kim et al. (2023) [41]	To evaluate a physical activity program on the self-efficacy of caregivers	Quasi-experimental design<br>Program: Physical Activity	n = 64 caregivers<br>Category: Informal<br>Age: 75 years<br>Gender: 20.31% men and 79.69% women<br>Region: South Korea (Asia)	It significantly increased caregivers' self-efficacy and reduced their burden, as well as improving health-related quality of life, physical function, and depressive symptoms. Strengthening self-efficacy acted as a protective mechanism against the demands of caregiving.	The study included only participants who were able to attend all sessions comprising the intervention.
Kim & Park (2023) [42]	To examine the relationship between self-esteem, resilience, and caregiver burden	Cross-sectional design	n = 109 caregivers<br>Category: Informal<br>Age: 35.88 years<br>Gender: 14.7% men and 85.3% women.<br>Region: South Korea (Asia)	Individuals with high levels of self-esteem and resilience were associated with lower overload. Self-esteem acted as a mediating factor that strengthened the impact of resilience in reducing perceived burden.	The sample was limited to caregivers of children with hematological and oncological diseases, which may reduce the generalizability.
Semere et al. (2023) [43]	To evaluate whether the nurse-led intervention (CONNECT) improves caregiver burden,	Randomized controlled design<br>Program: CONNECT	n = 441 caregivers<br>Category: Informal<br>Age: 61 years<br>Gender: 27% men and 73% women<br>Region: United States (America)	After the intervention, no significant changes were observed in overload or emotional symptoms independently. However, a statistically significant improvement in self-efficacy was evident, achieving a reduction in overload. Strengthening self-efficacy can act as a	The sample chosen was limited by several factors such as race, culture, socioeconomic status, etc., so there may be difficulties in generalizing the results.

	mood, and self-efficacy.			protective resource against the demands of caregiving, promoting a more adaptive coping strategy in the medium and long term. The intervention does not reduce overload on its own, but it can provide individuals with the skills to do so.	
Sun et al., (2023) [44]	To explore the level of burden on caregivers and the factors that influence it.	Controlled clinical trial	n = 100 caregivers<br>Category: Informal<br>Age: 30-60 years<br>Gender: 45% men and 55% women.<br>Region: China (Asia)	Self-efficacy was identified as a prerequisite for caregivers to exhibit better caregiving behaviors. Greater self-efficacy allows tasks to be tackled more effectively and with fewer negative emotions. Caregivers who had a high degree of self-efficacy experienced less overload.	NR
Yang et al., (2023) [45]	To explore the mediating role of self-efficacy and well-being in the relationship between caregiver burden and sleep quality.	Cross-sectional design	n = 325 caregivers<br>Category: Informal<br>Age: 51.42 years<br>Gender: 56.6% men and 43.4% women.<br>Region: China (Asia)	Self-efficacy and well-being partially mediated the relationship between burden and sleep quality. A higher degree of self-efficacy was associated with a lower negative impact of the burden.	When presenting a cross-sectional design, there may be difficulties in establishing causal relationships.
Banitalebi et al. (2022) [46]	To investigate the effect of a self-management program on the burden of care and self-efficacy in caregivers	Quasi-experimental design<br>Program: Self-management	n = 70 caregivers<br>Category: Informal<br>Age: 40.5 years<br>Gender: 42.9% men and 57.1% women<br>Region: Iran (Asia)	Self-management programs are an effective tool for reducing the burden of care and increasing self-efficacy. Improving self-management through the acquisition of coping skills, self-care, social support, and spiritual support promotes greater self-efficacy and reduces the burden of care.	Psychological problems and personal differences among caregivers are not taken into account, which could influence the generalization of the results.
Cao et al. (2022) [47]	To analyze caregiver overload, social	Cross-sectional design	n = 328 caregivers<br>Category: Informal<br>Age: 49.9 years	The analyses showed that self-efficacy, together with social support, acted as a key protective factor, significantly	The sample chosen was limited to one region of China.

	support, and self-efficacy		Gender: 42.1% men and 57.9% women<br>Region: China (Asia)	reducing the burden. This finding underscores that in a context of limited resources, boosting caregivers' confidence in their own abilities can be decisive in mitigating the emotional and physical impact of caregiving.	
Noel et al. (2022) [48]	To evaluate whether a virtual education program changes caregivers' confidence, self-efficacy, and burden.	Mixed design<br>Program: Virtual Educational Program	n = 134 caregivers<br>Category: Informal<br>Age: 18-65 years<br>Gender: 30% men and 70% women<br>Region: United States (America)	This program strengthened caregivers' confidence and self-efficacy. Results have shown that caregivers with greater confidence and self-efficacy have better health outcomes and reduced stress, burden, and depressive symptoms. Health professionals and health organizations should create virtual education programs for caregivers in rural and community settings.	The homogeneous sample chosen may limit the generalizability of the results.
Cheng et al. (2022) [8]	To examine the relationship between quality of life and caregiving burden and to assess the mediating role of self-esteem and psychological distress.	Cross-sectional design	n = 419 caregivers<br>Category: Informal<br>Age: 50.5 years<br>Gender: 45.3% men and 54.7% women<br>Region: Taiwan (Asia)	Self-esteem and psychological distress acted as mediating variables between the burden of care and caregivers' quality of life. Higher self-esteem was associated with lower perceived burden and better quality of life, while higher psychological distress increased the burden and reduced quality of life.	The cross-sectional design makes it difficult to establish causal relationships.
Schuit et al. (2022) [49]	To investigate the effect of Oncokompas and its effectiveness on caregiver burden,	Randomized clinical design<br>Program: OncoKompas	n = 58 caregivers<br>Category: Informal and formal<br>Age: 57 years<br>Gender: 33% men and 67% women	This study showed no effect of Oncokompas on caregiver burden or self-efficacy among caregivers of patients with incurable cancer.	The sample size was small, so the degree of change in the sample may have been influenced by this.

	self-efficacy, and quality of life.		Region: Netherlands (Europe)		
Yang et al. (2022) [14]	To examine the relationship between self-esteem and the demands of caregiving, coping, overload, and health in caregivers.	Cross-sectional design	n = 61 caregivers<br>Category: Informal<br>Age: 52.49 years<br>Gender: 57.4% men and 42.6% women<br>Region: China (Asia)	Higher self-esteem was associated with better coping, lower overload, and better physical and psychological health in caregivers.<br>Self-esteem acted as a mediator between caregiving demands and health outcomes.	The cross-sectional design makes it difficult to establish causal relationships.
Bruinsma et al. (2021) [50]	To evaluate a web-based intervention aimed at improving the psychological resources of caregivers.	Quasi-experimental design<br>Program: Partner in Balance	n = 27 caregivers<br>Category: Informal<br>Age: 64.5 years<br>Gender: 11.11% men and 89.89% women<br>Region: Netherlands (Europe)	The web-based intervention improved perceived self-efficacy, thereby reducing overload. In addition, participants reported greater confidence in their abilities, better coping, and a more positive perception of their role as caregivers.	The sample was small.
Rudelli et al. (2021) [51]	To analyze how the perception of the parental role influences self-efficacy, burden, and satisfaction with caregiving.	Cross-sectional design	n = 63 caregivers<br>Category: Informal<br>Age: 45.2 years<br>Gender: NR<br>Region: Switzerland (Europe)	A positive perception of the paternal role was associated with greater satisfaction in caregiving and higher levels of self-efficacy. Negative perceptions were associated with a greater burden. Self-efficacy was identified as a protective factor that mediates the relationship between role perception and overload.	The small sample size may make it difficult to generalize the results.
Ben–Pazi et al., (2020) [52]	To examine how parental self-efficacy moderates the relationship	Cross-sectional design	n = 70 caregivers<br>Category: Informal<br>Age: 43.77 years<br>Gender: 34.28% men and 65.72% women	Parents with high levels of self-efficacy experienced less overload. On the other hand, greater empathy was associated with a greater caregiver burden. Self-efficacy acts as a key moderator that	The sample chosen for the study is small.

	between empathy and overload.		Region: Israel (Asia)	reduces the negative impact of empathy on overload, promoting a more adaptive coping strategy.	
Leung et al. (2020) [53]	To evaluate the associations between social support and the mediating role of self-efficacy in caregiving on caregiver burden and patient quality of life.	Cross-sectional design	n = 225 caregivers<br>Category: Informal<br>Age: 57.1 years<br>Gender: 35.1% men and 64.9% women<br>Region: Hong Kong (Asia)	Self-efficacy in caregiving showed a negative correlation with caregiver burden and improved the patient's quality of life. Sources of social support were shown to be tools that moderate the associations between self-efficacy in caregiving, caregiver burden, and the patient's quality of life.	The cross-sectional design means that the results found need to be examined more thoroughly in longitudinal studies to establish temporal validity.
Rabiei et al., (2020) [54]	To evaluate the effect of a family-centered intervention on caregivers' self-efficacy.	Randomized clinical trial<br>Program: Family-Centered Intervention/Empowerment Program	n = 60 caregivers<br>Category: Informal<br>Age: 32-39 years<br>Gender: 15.6% men and 84.4% women<br>Region: Iran (Asia)	After the intervention, an increase in self-efficacy was identified, which reduced caregivers' perception of burden. The improvements were associated with knowledge management, coping skills, family communication, and caregiver empowerment.	The participants come from a single hospital, which may limit the generalizability of the findings.
Ramzani et al., (2019) [55]	To analyze the relationship between self-efficacy and perceived burden in caregivers	Cross-sectional design	n = 384 caregivers<br>Category: Informal<br>Age: 20-60 years<br>Gender: 39.9% men and 60.1% women<br>Region: Iran (Asia)	Self-efficacy showed a negative correlation with caregiver burden, i.e., the higher the self-efficacy, the lower the perceived burden. Strengthening self-efficacy may be a key strategy for reducing overload and improving caregivers' well-being.	The sample belongs to a context located in a single Iranian center, which limits the generalization of the results.
Richards et al. (2019) [56]	To evaluate the effect of a visual arts education program on the self-esteem and	Randomized clinical design<br>Program: Visual arts	n = 26 caregivers<br>Category: Informal<br>Age: 61.9 years<br>Gender: 19.3% men and 80.7% women	The visual arts intervention significantly improved the self-esteem of people with dementia and reduced the perceived burden on their caregivers.	The sample size is small, which could cause difficulties when generalizing the results.

	workload of caregivers.		Region: United States (America)		
Samia et al., (2019) [57]	To evaluate the effectiveness of a program in strengthening self-efficacy	Controlled trial<br>Program: Savvy Advanced	n = 100 caregivers<br>Category: Informal<br>Age: NR<br>Gender: NR<br>Region: United States (America)	The program improved caregivers' self-efficacy in managing challenging behaviors, problem solving, and stress management. Participants reported greater confidence in their abilities, less overload, and a better perception of well-being.	The study focused on experienced caregivers, which limits its generalizability to novice caregivers.
Yang et al. (2019) [58]	To verify how self-efficacy and social support are two mediating variables in caregiver overload, anxiety, and depression.	Mixed design	n = 112 caregivers<br>Category: Informal<br>Age: 20-60 years<br>Gender: 41.1% men and 58.9% women<br>Region: China (Asia)	Social support and self-efficacy are two variables that work together to reduce caregiver overload and depression.	Small sample
Boyacıoğlu & Kutlu (2017) [59]	Evaluate the effectiveness of a psychoeducational intervention on caregivers' psychological resources.	Randomized clinical trial	n = 64 caregivers<br>Category: Informal<br>Age: 49 years<br>Gender: 32% men and 68% women<br>Region: Turkey (Asia)	The psychoeducational intervention had a positive impact on self-efficacy in managing caregiving tasks and stress control, reducing overload. This strengthening of self-efficacy also promoted a better perception of competence and confidence in their role.	Study limited to a single cultural context, Turkey, which may make generalization difficult.
González Guerra et al. (2017) [60]	To analyze the factors that modulate resilience and overload	Descriptive design	n = 25 caregivers<br>Category: Informal<br>Age: 51-72 years<br>Gender: 12% men and 88% women<br>Region: Cuba (Americas)	Low self-esteem and emotional intelligence were associated with greater overload. These low levels of self-esteem also cause difficulties in coping self-efficacy when facing stressful situations, thus increasing overload.	NR
MacDougall & Steffen (2017) [61]	To analyze the ability of self-efficacy to control	Cross-sectional design	n = 158 caregivers<br>Category: Informal<br>Age: 50 years	Self-efficacy allows caregivers to maintain control over disturbing and negative thoughts, and is also a significant	NR

	disturbing thoughts, a predictor of emotional eating, stress, and depression.		Gender: Women<br>Region: United States (America)	predictor of emotional eating, overload, and depression.	
Yildiz et al. (2017) [62]	To determine levels of self-efficacy and caregiver burden	Cross-sectional design	n = 101 caregivers<br>Category: Informal<br>Age: 18-46 years<br>Gender: 39.7% men and 59.3% women<br>Region: Turkey (Asia)	The burden of caregiving showed a negative relationship with self-efficacy. Programs focused on self-efficacy levels should be developed to increase caregivers' capabilities. In addition, to reduce the burden and increase their self-efficacy, responsibilities can be shared with other family members, providing time for rest and recreation.	NR
Duggleby et al. (2016) [63]	To analyze the correlation between associated factors (gender identity, location, self-efficacy, overload) and quality of life.	Mixed design	n = 185 caregivers<br>Category: Informal<br>Age: 65 years<br>Gender: 16.8% men and 83.2% women<br>Region: Canada (Americas)	High levels of general self-efficacy in caregivers were associated with better mental health. Confidence in their ability to cope with situations allows them to better control thoughts related to overload (distress, depression). It is also positively related to physical health.	The majority of the sample was Caucasian, so there may be difficulty in generalizing the results.
Pablo-Santiago et al. (2016) [64]	To evaluate the relationship between caregiver burden and self-efficacy.	Cross-sectional, correlational, and longitudinal design	n = 109 caregivers<br>Category: Informal<br>Age: 55.1 years<br>Gender: 23.9% men and 76.1% women<br>Region: Mexico (America)	A negative correlation was found between self-efficacy and overload: the greater the self-efficacy, the lower the overload. Affective self-efficacy showed the strongest association. It was demonstrated that caregivers' perceptions need to be improved in areas such as maintaining their mood and stable social relationships	The cross-sectional design may make it difficult to establish causal relationships.

				with other people, not feeling stressed about caregiving, being able to make their patients laugh, encouraging them, and caring for them with love.	
Univer et al. (2016) [65]	To analyze the level of burden and self-efficacy among caregivers	Cross-sectional and descriptive design	n = 658 caregivers<br>Category: Informal<br>Age: 46.86 years<br>Gender: 16.60% men and 83.40% women<br>Region: Turkey (Asia)	The results showed a negative relationship between overload and self-efficacy in caregivers. On the other hand, caregivers' levels of self-efficacy significantly predicted the likelihood of caregivers being depressed.	NR
Weiss et al., (2016) [66]	To explore the levels of perceived self-efficacy in caregivers, as well as their predictors.	Cross-sectional design	n = 324 caregivers<br>Category: Informal<br>Age: 47.7 years<br>Gender: 6.2% men and 93.8% women<br>Region: Canada (Americas)	Greater parental self-efficacy was associated with less burden and greater coping capacity. Other variables such as social support and caregiver resilience were positively related to self-efficacy, while problematic behaviors in children reduced it. Self-efficacy is identified as a protective mechanism in parents' adaptation to caring for adolescents and adults with ASD.	When presenting a cross-sectional design, it is difficult to establish causal relationships.
Jennings et al. (2015) [67]	To analyze the role of the caregiver, depressive symptoms, and self-efficacy in managing problematic situations.	Cross-sectional design	n = 307 caregivers<br>Category: Informal<br>Age: NR<br>Gender: 33% men and 67% women<br>Region: United States (America)	It was observed that only one-third of all caregivers showed sufficient confidence (self-efficacy) to manage problems related to the disease, thus reporting high levels of overload. The analyses indicated that low self-efficacy is associated with greater overload and more intense depressive symptoms, especially in caregivers who care for patients with severe behavioral problems.	It has a cross-sectional design, so there may be difficulties in establishing causal relationships.

Durmaz & Okanlı (2014) [68]	To analyze the effect of self-efficacy levels on the burden of care	Descriptive design	n = 62 caregivers<br>Category: Informal<br>Age: 41.59 years<br>Gender: 54.8% men and 45.2% women<br>Region: Turkey (Asia)	The results showed that self-efficacy and overload were inversely related, such that caregivers who were more confident in their own abilities to cope with the demands of caregiving perceived less burden. Self-efficacy was identified as a protective resource against the negative impact of caregiving on mental health.	NR
Shankar et al. (2014) [69]	To examine the perceived burden on caregivers at the time of hospitalization and associated factors	Cross-sectional design	n = 495 caregivers<br>Category: Informal<br>Age: 61 years<br>Gender: Women<br>Region: United States (America)	A higher degree of self-efficacy was associated with lower overload. Those caregivers who also had little confidence in their abilities had higher levels of stress and burden. Greater self-efficacy perceived by caregivers was associated with lower overload.	When presenting a cross-sectional design, it is difficult to establish causal relationships.
Zhang et al. (2014) [70]	Explore the mediating role of caregiver self-efficacy between caregiver subjective burden, behavioral and psychological symptoms of dementia, and social support.	Cross-sectional design	n = 137 caregivers<br>Category: Informal<br>Age: 69.9 years<br>Gender: NR<br>Region: China (Asia)	Caregiver self-efficacy has a positive impact by reducing subjective overload and the behavioral and psychological symptoms of dementia. It also acts as a moderator between social support and perceived burden, so strengthening caregiver confidence can mitigate the emotional impact of caregiving, especially in highly demanding contexts.	The small sample size makes it difficult to explore more complex relationships between the variables under study.
Gallagher et al. (2011) [71]	To analyze how self-efficacy manages symptoms of overload and	Cross-sectional design	n = 85 caregivers<br>Category: Informal<br>Age: 63.3 years<br>Gender: 42.9% men and 57.1% women<br>Region: Ireland (Europe)	Caregivers who showed greater self-efficacy in managing dementia symptoms experienced less burden and depression, even when factors such as neuroticism and the clinical severity of the patient were taken into account. When cognitive	The cross-sectional design of the study may make it difficult to draw causal conclusions.

	depression in caregivers			impairment was moderate to severe, self-efficacy acted as a mediator between the patient's symptoms and the caregiver's distress, highlighting its protective role in more demanding contexts.	
Merluzzi et al., (2011) [72]	To evaluate self-efficacy and self-care and their impact on reducing stress and overload.	Cross-sectional design	n = 133 caregivers<br>Category: Informal<br>Age: 57<br>Gender: 28% men and 72% women<br>Region: United States (America)	Caregivers with greater self-efficacy in self-care and emotional support showed less overload and stress. A low perception of efficacy was associated with greater symptoms related to depression and lower well-being.	When presenting a cross-sectional design, there are difficulties in establishing causal relationships.
Gonyea et al. (2005) [73]	To explore how self-efficacy and depressive symptoms influence the subjective experience of caregiver burden.	Cross-sectional design	n = 80 caregivers<br>Category: Informal<br>Age: 64.8 years<br>Gender: 36% men and 64% women<br>Region: United States (America)	Caregivers with higher self-efficacy experienced less overload, even when faced with high levels of caregiving demands. Self-efficacy proved to be a protective mechanism against negative perceptions of the role. Depressive symptoms significantly increased the burden. Self-efficacy could mitigate the impact of depression on the caregiving experience.	The cross-sectional design makes it difficult to establish causal relationships.

Note: AFD, Authoritarian filial pretty; ASD, Autism Spectrum Disorder.; NR, Not reported.

Table S2. PRISMA Checklist.

Section and Topic	Item #	Checklist Item	Location Where Item is Reported
**TITLE**			
Title	1	Identify the report as a systematic review.	1
**ABSTRACT**			
Abstract	2	See the PRISMA 2020 for Abstracts checklist.	1
**INTRODUCTION**			
Rationale	3	Describe the rationale for the review in the context of existing knowledge.	2
Objectives	4	Provide an explicit statement of the objective(s) or question(s) the review addresses.	3
**METHODS**			
Eligibility criteria	5	Specify the inclusion and exclusion criteria for the review and how studies were grouped for the syntheses.	6
Information sources	6	Specify all databases, registers, websites, organisations, reference lists and other sources searched or consulted to identify studies. Specify the date when each source was last searched or consulted.	4
Search strategy	7	Present the full search strategies for all databases, registers and websites, including any filters and limits used.	5
Selection process	8	Specify the methods used to decide whether a study met the inclusion criteria of the review, including how many reviewers screened each record and each report retrieved, whether they worked independently, and if applicable, details of automation tools used in the process.	6
Data collection process	9	Specify the methods used to collect data from reports, including how many reviewers collected data from each report, whether they worked independently, any processes for obtaining or confirming data from study investigators, and if applicable, details of automation tools used in the process.	7
Data items	10a	List and define all outcomes for which data were sought. Specify whether all results that were compatible with each outcome domain in each study were sought (e.g., for all measures, time points, analyses), and if not, the methods used to decide which results to collect.	3
	10b	List and define all other variables for which data were sought (e.g., participant and intervention characteristics, funding sources). Describe any assumptions made about any missing or unclear information.	4
Study risk of bias assessment	11	Specify the methods used to assess risk of bias in the included studies, including details of the tool(s) used, how many reviewers assessed each study and whether they worked independently, and if applicable, details of automation tools used in the process.	7
Effect measures	12	Specify for each outcome the effect measure(s) (e.g., risk ratio, mean difference) used in the synthesis or presentation of results.	NR

Synthesis methods	13a	Describe the processes used to decide which studies were eligible for each synthesis (e.g., tabulating the study intervention characteristics and comparing against the planned groups for each synthesis (item #5)).	7
	13b	Describe any methods required to prepare the data for presentation or synthesis, such as handling of missing summary statistics, or data conversions.	7
	13c	Describe any methods used to tabulate or visually display results of individual studies and syntheses.	10
	13d	Describe any methods used to synthesize results and provide a rationale for the choice(s). If meta-analysis was performed, describe the model(s), method(s) to identify the presence and extent of statistical heterogeneity, and software package(s) used.	10
	13e	Describe any methods used to explore possible causes of heterogeneity among study results (e.g., subgroup analysis, meta-regression).	17
	13f	Describe any sensitivity analyses conducted to assess robustness of the synthesized results.	NR
Reporting bias assessment	14	Describe any methods used to assess risk of bias due to missing results in a synthesis (arising from reporting biases).	NR
Certainty assessment	15	Describe any methods used to assess certainty (or confidence) in the body of evidence for an outcome.	7
**RESULTS**			
Study selection	16a	Describe the results of the search and selection process, from the number of records identified in the search to the number of studies included in the review, ideally using a flow diagram.	6
	16b	Cite studies that might appear to meet the inclusion criteria, but which were excluded, and explain why they were excluded.	7
Study characteristics	17	Cite each included study and present its characteristics.	19
Risk of bias in studies	18	Present assessments of risk of bias for each included study.	NR
Results of individual studies	19	For all outcomes, present, for each study: (a) summary statistics for each group (where appropriate) and (b) an effect estimate and its precision (e.g., confidence/credible interval), ideally using structured tables or plots.	11
Results of syntheses	20a	For each synthesis, briefly summarise the characteristics and risk of bias among contributing studies.	19
	20b	Present results of all statistical syntheses conducted. If meta-analysis was done, present for each the summary estimate and its precision (e.g., confidence/credible interval) and measures of statistical heterogeneity. If comparing groups, describe the direction of the effect.	14

	20c	Present results of all investigations of possible causes of heterogeneity among study results.	11
	20d	Present results of all sensitivity analyses conducted to assess the robustness of the synthesized results.	NR
Reporting biases	21	Present assessments of risk of bias due to missing results (arising from reporting biases) for each synthesis assessed.	14
Certainty of evidence	22	Present assessments of certainty (or confidence) in the body of evidence for each outcome assessed.	NR
**DISCUSSION**			
Discussion	23a	Provide a general interpretation of the results in the context of other evidence.	15
	23b	Discuss any limitations of the evidence included in the review.	16
	23c	Discuss any limitations of the review processes used.	16
	23d	Discuss implications of the results for practice, policy, and future research.	16
**OTHER INFORMATION**			
Registration and protocol	24a	Provide registration information for the review, including register name and registration number, or state that the review was not registered.	3
	24b	Indicate where the review protocol can be accessed, or state that a protocol was not prepared.	3
	24c	Describe and explain any amendments to information provided at registration or in the protocol.	3
Support	25	Describe sources of financial or non-financial support for the review, and the role of the funders or sponsors in the review.	34
Competing interests	26	Declare any competing interests of review authors.	34
Availability of data, code and other materials	27	Report which of the following are publicly available and where they can be found: template data collection forms; data extracted from included studies; data used for all analyses; analytic code; any other materials used in the review.	34
